# Camera Traps: A Novel Method to Estimate Numbers of Nesting Sea Turtles

**DOI:** 10.1002/ece3.72138

**Published:** 2025-09-15

**Authors:** Holly J. Stokes, Graeme C. Hays, Kimberley L. Stokes, Nicole Esteban

**Affiliations:** ^1^ Department of Biosciences Swansea University Swansea UK; ^2^ Deakin Marine Research and Innovation Centre, School of Life and Environmental Sciences Deakin University Geelong Victoria Australia

**Keywords:** endangered species, marine megafauna, marine protected area (MPA), marine turtle, population monitoring, remote monitoring, sampling

## Abstract

1. Abundance estimates are difficult to obtain for many animal groups, yet essential for endangered species management and conservation. For sea turtles, estimates are made from ground counts of nesting females, tracks, and nests, but these are challenging at remote locations. Here we explore the applicability of using camera traps to monitor and estimate sea turtle nesting tracks at a green turtle ( *Chelonia mydas* ) rookery in the Western Indian Ocean.
2. Camera traps (*n =* 13) were deployed to photograph turtle tracks daily along a 2.8 km beach in Diego Garcia, Chagos Archipelago in 2021 and 2022. Foot patrol surveys were conducted in April and May 2021 and August 2022 (14, 13 and 20 days, respectively). Track counts were compared from both methods to validate the use of cameras.
3. From foot patrol surveys, we observed an increase in track counts around neap tides (mean ± SD: 5.0 ± 4.0 tracks per day; *n =* 131 tracks) compared to spring tides (2.4 ± 1.8 tracks per day; *n =* 51 tracks). Mean track longevity was similar during neap (2.9 ± 2.0 days; *n =* 39 tracks) and spring tides (2.7 ± 2.6 days; *n =* 20 tracks). Mean daily track counts were comparable during neap tides (camera traps: 7.3 ± 12.9 tracks *cf.* patrols: 5.0 ± 4.0 tracks) and across the tidal cycle (camera traps: 5.5 ± 13.1 tracks *cf.* patrols: 3.9 ± 3.4 tracks). Using simulated data, we found track count variability decreased in a power‐law relationship with increasing coverage by cameras. The disparity in track counts between methods would likely decrease if beach coverage increased from 5% to 20%.
4. Camera traps provide a complementary tool to fill data gaps at remote sites that would otherwise have little to no assessments. Furthermore, the increased temporal coverage from cameras can help identify changes in nesting phenology and trends in nesting numbers.

## Graphical abstract

We compared sea turtle track counts from camera traps with those from simultaneous foot patrol surveys. Our findings support the use of camera traps as a complementary tool to fill data gaps at remote sites that would otherwise receive little to no assessment. Furthermore, the increased temporal coverage from camera traps can help identify changes in nesting phenology and trends in nesting numbers.

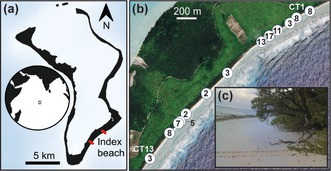

## 1 Introduction

Population abundance estimates are essential to make informed and effective management decisions (Nichols 2014), yet accurate estimates are difficult to obtain for many animal groups that are elusive or rare (McDonald 2004). Many studies rely on capture‐mark‐recapture (Labonne and Gaudin 2005), ground surveys (Udevitz et al. 2005), or aerial counts (McCarthy et al. 2022), which can be labour‐intensive, expensive, or logistically challenging. Additionally, capturing animals can influence sampling and potentially bias results (Fieberg et al. 2015). Hence, non‐invasive techniques that simultaneously reduce effort and cost are of interest (Pauli et al. 2010). Populations are often assessed using a single method, but the benefit of combining techniques to complement and enhance data quality is increasingly acknowledged in conservation (Zwerts et al. 2021) to create a broader perspective and increase spatial and temporal coverage (Rahman and Rahman 2021).

Marine megafauna populations are often particularly difficult to assess given that most of their time is spent offshore (Hays et al. 2016). However, abundance estimates are possible for some species when individuals come ashore to breed or rest (e.g., seals; Southwell et al. 2008 and turtles; Lasala et al. 2023). For sea turtles, ideally nesting beaches would be surveyed frequently and systematically to intercept all nesting females, but this is not possible at many sites with limited resources or remote locations, and so in these cases, estimates are made from infrequent track and nest counts (Whiting et al. 2013).

Camera traps are used to answer a wide range of ecological questions (Hamel et al. 2012). Cameras can remain in the field for months, reducing resources and disturbance (McCallum 2013), and operate day and night in harsh conditions (Rowcliffe et al. 2014). While initially expensive, camera traps are economical long‐term as they can be used for multiple seasons and species (Welbourne et al. 2020). Camera trap research often focuses on elusive terrestrial mammals using the trigger function to capture animals passing by (Fisher et al. 2011; Lyet et al. 2023), sometimes identifying individuals, such as tigers, by their unique stripe pattern (Royle et al. 2009).

Several studies have validated the use of remote techniques for sea turtle research, for example, stereo‐video cameras to remotely measure body size (Piacenza et al. 2022) and satellite imagery to assess nesting activity (Casale and Ceriani 2019). Camera traps have been used specifically to address a key threat to sea turtles: predation (Fuentes et al. 2023). Recent studies have identified predators of nesting turtles, for example, Jaguar ( *Panthera onca* ) in Costa Rica (Fonseca et al. 2020), invasive rat ( *Rattus rattus* ) predation of turtle hatchlings in French Polynesia (Gronwald et al. 2019) and nest predation by yellow spotted goannas ( *Varanus panoptes* ) and red foxes ( *Vulpes vulpes* ) in Australia (Lei and Booth 2017). Predator behaviour patterns (Guilder et al. 2015) and strategies for nest protection (Lovemore et al. 2020) have also been investigated using camera traps. There are no published studies on the use of camera traps for estimates of nesting turtle numbers.

Given the increase in camera trap use and the need to assess sea turtle populations at remote (e.g., Chandeleur Islands; Lamont et al. 2023) or dangerous locations (e.g., presence of large predators; Whiting and Whiting 2011) with increased temporal coverage, we investigate how camera traps can be used to estimate numbers of nesting turtles at a key green turtle ( *Chelonia mydas* ) nesting site within a Marine Protected Area in the Western Indian Ocean. Additionally, we use simulated data to determine suitable camera trap coverage, which could be applied to nesting sites with different beach lengths and track density. Further, we show how camera traps can be used to assess inter‐annual variation, including temporal shifts in nesting seasons as a potential result of climate change.

## 2 Materials and Methods

### 2.1 Study Area

**FIGURE 1:**
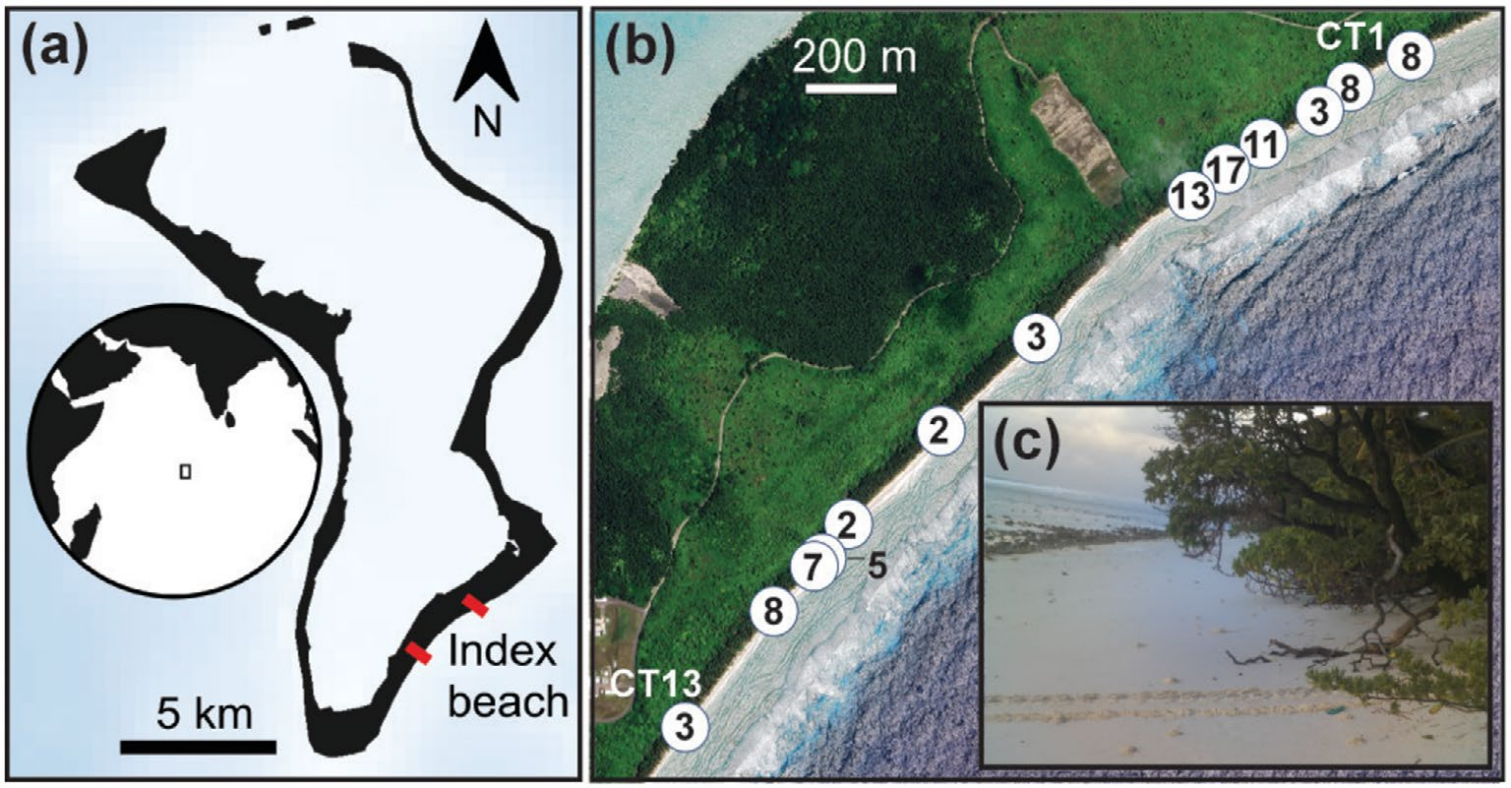
(a) Diego Garcia and inset map showing the location of the Chagos Archipelago (black rectangle). The study took place on the Index Beach (between red lines) on the southeast of Diego Garcia (source: GEBCO, 2021). (b) Camera trap locations from camera trap (CT) 1 to 13 are indicated by white circles. Numbers represent the total track count from each camera trap between April and September 2021 and 2022 (Basemap Google satellite imagery sourced through QGIS3). (c) Camera trap image of a green turtle ( *Chelonia mydas* ) track.

Diego Garcia (7.42° S, 72.45° E) is the largest and only inhabited island in the Chagos Archipelago and has 72.1 km of coastline, of which 40.5 km (56%) is categorised as suitable nesting habitat. Our study was undertaken along a 2.8 km stretch of beach (Index Beach) identified as one of the highest nesting density areas (Figure 1a). In the Chagos Archipelago, green turtles nest year‐round, mostly between June and October, with a peak in August, whilst hawksbills ( *Eretmochelys imbricata* ) mainly nest between October and February, with a peak in December (Mortimer et al. 2020).

### 2.2 Foot Patrol Surveys and Track Counts

Turtle tracks, defined as the imprint a turtle leaves in the sand, were counted during foot patrol surveys. The surveys were conducted during the months of April and May 2021 and August 2022 (14, 13 and 20 survey days within each month, respectively). For every survey, all tracks were counted, and a line was drawn through the track to avoid double counting. Mean track width was recorded from three measurements using a flexible tape measure and used along with track characteristics to identify species. Green turtles leave a symmetrical track (usually > 100 cm wide) in the sand, while hawksbills leave an asymmetrical track (typically < 95 cm; see Pritchard and Mortimer 1999 and Mortimer et al. 2020). Mean track count per day was calculated using the number of fresh tracks recorded on survey days (*n =* 47 days).

### 2.3 Tidal Influence on Track Count

**FIGURE 2:**
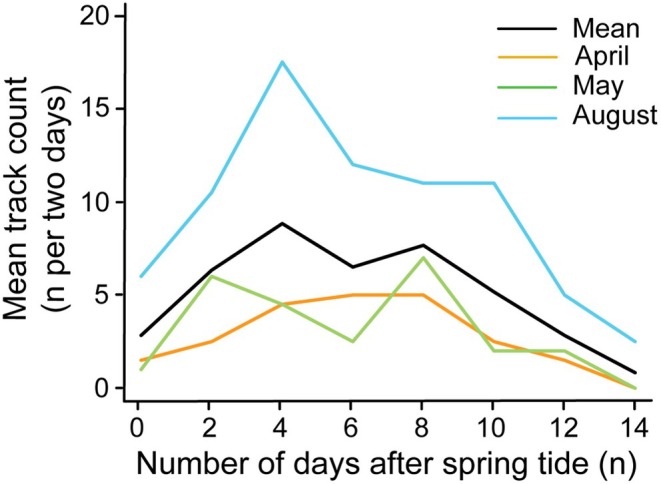
Track counts were higher during days around neap tide (Days 4–11) compared to days around spring tide (Days 0–3 and 12–14) shown from foot patrol surveys in April and May 2021, August 2022. Spring tide (Day 0) is the highest tide with the greatest tidal range on or after the most recent full or new moon. Across some tidal cycles, track counts show a bimodal distribution with a peak in counts either side of the neap tide (~Day 7).

To understand the influence of tides on track counts, we assessed the number of track counts from foot patrol surveys along the tidal cycle as the number of days after spring tide, spring tide being the highest tide with the greatest tidal range on or after the most recent full or new moon. Days of the tidal cycle were split into days around spring tide (Days 0–3; 12–14) and neap tide (Days 4–11). We used one full tidal cycle in the middle of each month. On the days where there was no survey the day before a survey day, any tracks visibly > 24 h old were recorded on the ‘no survey day’ and any tracks < 24 h were recorded on the survey day. If there were 2 days between surveys, then tracks > 24 h were split evenly across the 2 days or if there was an odd number then more tracks were added to the day before the survey (e.g., if there were three visibly old tracks and 2 days since the last survey, two tracks were assigned to the previous day and one to the day before that). In the instance of 3 days between survey days over neap tides, the same principle was used, for example, if there were four tracks to cover 3 days of no surveys then two tracks were assigned to the day before the survey and one track to 2 days before the survey and one track to 3 days before the survey. Mean track count was calculated across 2 days of the tidal cycle (i.e., mean of Day 0 and Day 1, Day 2 and Day 3; Figure 2).

### 2.4 Camera Trap Survey Design and Settings

Camera traps (Apeman H70; *n =* 13) were attached to tree trunks or large branches (e.g., *Tournefortia argentea* ; Appendix S1: Figure A1a) lining the nesting beach. Cameras were positioned to be equally spaced along the survey area as far as possible and to capture the length of the beach. Camera placement was affected by (a) vegetation available for attachment and avoidance of (b) vegetation obstructing the camera's field of view and (c) overlap of images (Appendix S1: Figure A1b). There was equal potential for cameras to be placed in areas of higher or lower nesting activity. The distance observed from each camera trap was measured by creating ‘turtle tracks’ in the sand every 5 m until the tracks were no longer visible (range from a single camera trap = 5–30 m; Appendix S1: Table A1). The number of ‘turtle tracks’ visible from each camera was counted both in person and from images (Appendix S1: Figure A1b). Estimated distances of beach coverage differed from each camera (e.g., due to variability in vegetation obstruction along the beach) and from the same camera over time (e.g., due to the camera falling slightly and altering the angle and view from the camera; Table A1; for more detail see Appendix S1).

Trigger settings were disabled, and camera traps were set to take daily images on time‐lapse with 30 min intervals between 0700 and 0800 h. In 2022, ten of the cameras were set to take images every 30 s from 0700 to 0830 h starting July 13.

### 2.5 Camera Trap Image Processing and Track Count

Images were processed using a 4 k monitor and data recorded from image analysis (e.g., Figure 1c) included camera trap ID, date, track count, track longevity and estimated distance observed from the camera trap. An emergence was recorded if there was a single track or an up‐ and down‐track on the beach. Reasons for a single track in the camera trap image could be because (a) the turtle had not returned to the sea before the photographs were taken or (b) the down track was out of the camera trap field of view (e.g., behind the camera trap). We assumed all tracks observed in camera trap images were from green turtles as surveys took place outside of the peak hawksbill nesting season, and although possible, very few hawksbills nest in the Chagos Archipelago during this time of the year (see Mortimer et al. 2020). Additionally, tracks were allocated to species and confirmed during foot patrol surveys that were conducted simultaneously with camera trap surveys.

For each day during camera trap surveys in April and May 2021 and August 2022 (*n =* 91 days), raw camera trap track counts were extrapolated to the whole beach, assuming that the density of tracks on the camera‐monitored sections reflected the density across the entire beach using the following formula:

$$\left(\text{Total beach distance}/\text{distance covered}\;\mathrm{by}\;\text{camera traps}\right)\times \mathrm{raw}\;\text{camera trap track counts}$$

To calculate track longevity (the number of days a turtle track persists on the beach), we used camera trap images from April to September 2021 and July to August 2022, which coincided with our frequent (April and May 2021 and August 2022) and infrequent foot patrol surveys on the Index Beach around the green turtle nesting season. Track longevity was recorded for each track unless the track was already present on the first day cameras started recording, or if the camera stopped working when a track was still present. Longevity was also not counted if the vegetation line where the track enters the vegetation was out of view from the camera trap, as the track could remain just in front of the vegetation line, but this would not be captured in the image. Mean track longevity was calculated across the full tidal cycle as well as separately for neap and spring tide.

During image processing, it was evident that the total observable distance in images from some camera traps had changed over time. We therefore reanalysed and estimated new distances from each camera trap using images with marked distances (e.g., Appendix S1: Figure A1b).

### 2.6 Simulation to Assess Optimal Beach Coverage by Camera Traps

We assessed how the extent of beach coverage by camera traps might be expected to affect the confidence in the estimate of the mean number of tracks per day. To do this, we ran a simulation parameterised by the length and typical number of tracks on our study beach. We assumed a beach length of 2.8 km and that the mean number of tracks per day was six. Then, for each 1 m section of beach for each day, we randomly picked if that 1 m would include a track from a binomial distribution with the probabilities of no track (*p*) and a track (*q*), being *p* = 0.9978572 and *q* = 0.0021428. Then, we randomly selected a percentage of the beach surveyed by camera traps and assessed how many of the tracks would be captured by the cameras each day. We ran the model for 90 days and worked out the mean number of tracks counted by the cameras for each of those 90 days, and then extrapolated up to the mean number of tracks for the whole beach. For each value of beach coverage by the cameras, we ran 100 simulations of 90 days each and then, from those 100 simulations, worked out the standard deviation (SD) of the estimated track count for the entire beach. We varied the percentage of the beach covered by the cameras (*n =* 13) from 2% to 40%.

### 2.7 Data Analyses

The relationship between camera trap beach coverage and track count variability was explored using linear modelling on log‐transformed data (Appendix S2: Figure A2). Model coefficients were back‐transformed for plotting on linear scales. A *t*‐test was used to test the significance between the model simulated mean track counts and the assumed mean number of six tracks per day. To compare camera trap track counts across the Index Beach, we ran a Kruskal‐Wallis rank‐sum test and a post hoc Dunn's test with Benjamini‐Hochberg (FDR) correction for pairwise comparisons of track counts between cameras. All plots were created, and statistical analyses conducted in R (R Core Team 2024; version 4.4.2). Data are presented as mean ± SD.

## 3 Results

Across 91 camera trap survey days during April and May 2021 and August 2022 (when camera trap and foot patrol surveys were running parallel), the mean number of cameras working was 11.0 per day (±2.8, range: 2–13 per day) resulting in a mean of 5.0% (±1.3, range: 0.2%–5.9%) or 124.9 m (±35.5, range: 5–165 m) of the Index Beach surveyed by camera traps each day. The mean number of cameras not working was 2.0 per day (±2.8, range: 0–11 per day) due to fog on the camera lens (0.3 ± 0.6, range: 0–2 per day), the camera falling and pointing downwards (0.6 ± 0.8, range: 0–2 per day), or because the camera had stopped working most often due to reaching memory card storage capacity when cameras were set to take images every 30 s (1.1 ± 2.9, range: 0–11 per day).

### 3.1 Tidal Influence on Nesting Emergences

From foot patrol surveys, we observed a bimodal distribution of tracks with peaks either side of neap tide across some tidal cycles (May 2021 and August 2022, Figure 2). Mean track count was 5.0 per day (±4.0; *n =* 131 tracks) over neap tides, which was higher than 2.4 per day (±1.8; *n =* 51 tracks) during spring tides (Figure 2).

### 3.2 Tidal Influence on Track Longevity

Mean track longevity was similar during neap tides (2.9 ± 2.0 days, range: 0–8 days, *n =* 39 tracks), spring tides (2.7 ± 2.6 days, range: 0–9 days; *n =* 20 tracks), and across the full tidal cycle (2.8 ± 2.2 days, range: 0–9 days, *n =* 59 tracks).

### 3.3 Track Distribution

Tracks were not distributed evenly across the Index Beach; we found a significant difference in track counts from the 13 cameras positioned along the length of the beach (*X* ^2^_(12)_ = 34.61; *p* < 0.001). Cameras 4, 5 and 6 recorded the highest overall track counts (Figure 1b), with camera trap 5 (*n =* 17 tracks) having a significantly higher track count than five other cameras spread along the beach (cameras 3, 7, 8, 9 and 13, range: 2–3 tracks, *p* < 0.05).

### 3.4 Foot Patrol and Camera Trap Track Count Comparison

We compared track counts from foot patrols and camera traps conducted only during neap tides, to align with standard foot patrols most often completed at this site, and across the full tidal cycle. Across the whole tidal cycle, from fresh tracks recorded during foot patrol survey days (*n =* 47 days) we recorded 182 tracks, of which 33 tracks were recorded in April 2021, 30 tracks in May 2021 and 119 tracks in August 2022. Across the 47 days (full tidal cycle) the mean number of tracks was 3.9 per day (±3.4, range: 0–15 per day, *n =* 182 tracks), whereas during neap tides (26 days) the mean number of tracks was 5.0 per day (±4.0, range: 0–15 per day, *n =* 131 tracks). All tracks were from green turtles.

From camera traps, a total of 21 tracks were observed between April and May 2021 and August 2022. For each day (*n =* 91 days) across the full tidal cycle, raw camera trap track counts were extrapolated to the whole Index Beach, and the mean track count was 5.5 per day (±13.1, range: 0–80 per day; *n =* 499.9 tracks). When solely looking at track counts during neap tides (*n =* 49 days) the mean track count was 7.3 per day (±12.9; range: 0–50.9 per day, *n =* 356.0 tracks).

### 3.5 Camera Settings

From functioning cameras (excluding cameras that had stopped working or had fallen) across 91 days, we obtained 1029 photos with a clear image on 1002 occasions (97%). Cameras operated for a maximum of 12 months when set to capture images every 30 min between 0700 and 0800 h each day. When the camera trap time‐lapse interval was set to 30 s between 0700 and 0830 h in July and August 2022, cameras stopped working due to reaching memory card storage capacity after 36 to 40 days (37.6 ± 1.4 days; *n =* 9 cameras, battery level still high), with each camera capturing 180 images per day, totaling a minimum of 6480 images over 36 days before reaching storage capacity. Given these findings, theoretically an image could be captured every 2 h for 12 months in future studies.

### 3.6 Simulation to Assess Optimal Beach Coverage by Camera Traps

**FIGURE 3:**
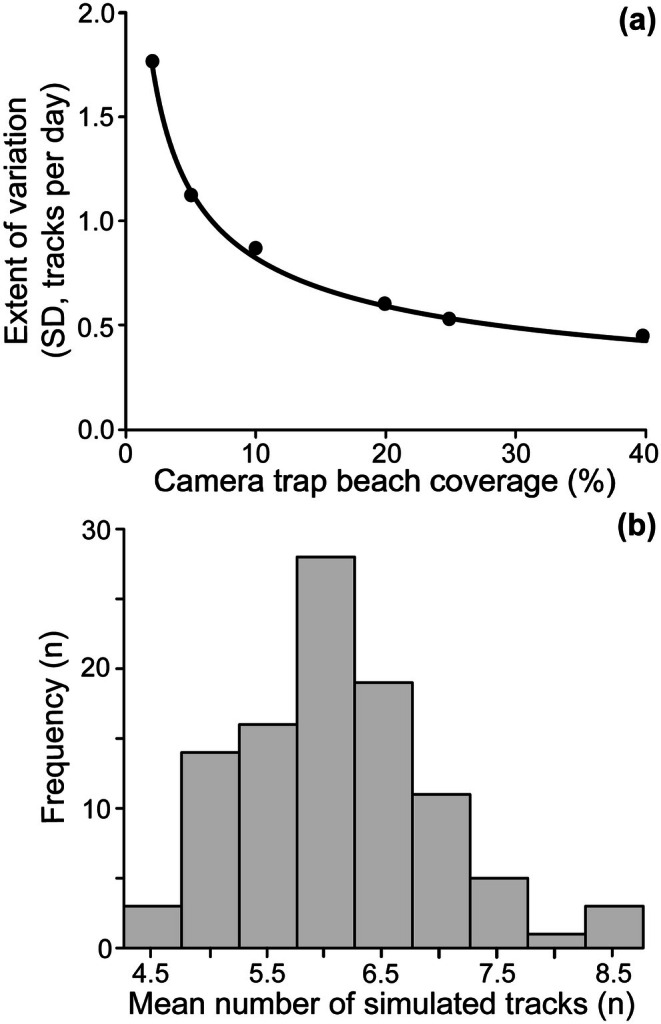
Camera trap beach coverage influences the reliability of track counts. (a) The standard deviation for the estimated mean number of tracks per day over a 90‐day simulation with different extents of beach coverage by cameras. Track count variability decreased in a power‐law relationship as beach coverage increased (SD = 2.477475/Coverage^0.47902^; black line). (b) An example frequency distribution for the mean track count per day over a 90‐day simulation, repeated 100 times. In this case 10% of the beach was covered by cameras, and the mean ± SD track count was 6 ± 0.86.

We explored the effect of increasing the number of camera traps and found a significant decrease in track count variability (SD, tracks per day) as the extent of beach coverage by cameras increased. This decrease was best described by a power‐law model (Figure 3a); log transformed variables showed a strong straight‐line relationship (*R* ^2^ = 0.99; *F*_1,4_ = 1443; *p* < 0.0001; Appendix S2: Figure A2). For example, if camera traps covered 10% of the beach, while the mean number of tracks for the 90‐day simulations (6.08) was not significantly different from 6 (*t*_99_ = 0.95; *p* = 0.34), the SD was 0.8618, that is, 95% of the estimated mean daily number of tracks were between 4.393 and 7.771 tracks per day (Figure 3b). Optimal beach coverage would be around 20% (Figure 3a) and so the coverage (5%) at our study site was low. The maximum distance observed from a single camera at our study site was 30 m. If we were to place cameras along the beach with minimal obstructions so each camera could capture 30 m, then we could assume that 18.6, rounded to 20 camera traps would cover 20% (in this case 560 m) of the 2800 m Index Beach.

## 4 Discussion

By applying the novel approach of camera traps alongside foot patrol surveys on sea turtle nesting beaches, we demonstrate how camera traps can be used to count tracks and determine track longevity. This finding is noteworthy given that many sea turtle nesting datasets are temporally fragmented (Omeyer et al. 2022) due to varying efforts within and across years and locations, particularly at remote or extensive sites (Shimada et al. 2021). Camera trap surveys can fill this temporal gap through the collection of data in the field long‐term. Moreover, as nesting numbers (Broderick et al. 2003; Hays et al. 2024) and beach length vary (Kikukawa et al. 2001), we explain how camera traps could be applied to other sea turtle nesting sites around the world.

We found that track counts from foot patrol surveys and camera trap surveys were comparable, validating the use of camera traps. Digitised surveys are increasingly trialled at sea turtle nesting sites (e.g., aerial photogrammetry surveys; Tucker et al. 2021). Like our findings, in the Pilbara region of Western Australia, a significant positive relationship was found between track counts from photographs taken from an aeroplane and ground‐based surveys, suggesting aerial photogrammetry as an effective method to collect nesting turtle distribution and abundance data (Fossette et al. 2021). Though our findings from camera traps and foot patrol surveys were comparable, camera trap counts were higher, likely due to unintentional camera placement at potential “hotspots” or underestimating the distance covered by camera traps. Total distance observed from cameras varied for each camera and the same camera over time, an important factor to consider, as the accuracy of the estimated distance of the beach covered by cameras can greatly impact the track count estimate. The disparity between track counts from foot patrols and cameras was likely due to low coverage of the Index Beach (5%) and would be less if 20% coverage was attained at this study site. Track counts between the two methods were within the same order of magnitude, making camera trap counts a reliable proxy for assessing population trends. In a similar manner, several well‐designed studies have assessed trends in sea turtle populations from foot patrol time series data (Weber et al. 2014; Medeiros et al. 2022). Sea turtles do not generally breed every year and are likely to skip years of unfavourable environmental conditions when food availability is low (Broderick et al. 2003) and so there is often substantial inter‐annual variability in sea turtle nesting numbers, as much as 60‐fold between successive years (Hays et al. 2022), that influences trend assessments. Given the naturally high inter‐annual variability in nesting numbers, the difference between camera trap and foot patrol track counts is negligible. For populations where inter‐annual variability greatly influences the ability to detect trends, data across two or three consecutive years can be averaged (Mazaris et al. 2017) and so the same could be applied for data obtained from camera traps.

Three key factors considered for optimal experimental design were (i) the number of camera traps; (ii) the duration of monitoring; and (iii) timing of surveys (Kays et al. 2020). Firstly, to assess the number of camera traps needed for optimal coverage, we investigated track count variability in relation to sampling effort using simulated data and found that by increasing the number of camera traps along the nesting beach, track count variability decreased in a power‐law relationship with beach coverage. This means there are large gains initially while increasing % beach coverage, and this quickly changes to diminishing returns after a certain amount of beach coverage has been reached. Similarly, Luo et al. (2020) found variance in estimates decreased when increasing the number of camera traps and monitoring duration. At our study site, mean beach coverage per day was 5% due to camera lens fogging, cameras falling or vegetation obstruction, and reaching memory card storage capacity. For future work, 20% (in this case 560 m) beach coverage would be optimal for beaches with relatively low track density, and so if each camera was set to capture 30 m each of the beach, then we would recommend 20 camera traps. Our model can be extended to ensure coverage is optimal on beaches of differing lengths and nesting densities. Many sea turtle studies include power analyses to determine the minimum temporal sampling effort needed to detect similar population trends when compared to continuous sampling (Sims et al. 2008; Girondot 2017; Whiting et al. 2020, 2021). Whiting et al. (2021) found that coverage of 5% provided relatively accurate estimates of annual nesting activity, highlighting that annual studies, even with low coverage, are important to estimate sea turtle abundance. To detect terrestrial animals in an enclosed park with camera traps, the effort (i.e., number of camera traps and duration) needed to obtain a sufficient sample size varied by density and range (Rowcliffe et al. 2008). For long‐term monitoring of sea turtle nesting beaches in Northwest Australia, aerial surveys are ideal for higher density nesting, whilst Traditional Ecological Knowledge is vital to understand sparse and infrequent nesting (Tucker et al. 2021). In our case, camera traps are likely best suited to beaches that support low to moderate nesting activity, as high‐density nesting with overlapping tracks could decrease the accuracy of track counts.

Secondly, it is noteworthy that camera traps can operate in the field for up to one year. With that in mind, in the Chagos Archipelago, 86% of hawksbill nesting activity occurs between October and February, and whilst green turtles nest year‐round, 64% of nesting occurs between June and October, and so cameras can be set with no servicing to monitor the peak nesting season for both species (Mortimer et al. 2020). Nesting season duration can vary year on year (Mrosovsky et al. 1984), by species (Mortimer et al. 2020) and location (Dewald and Pike 2013) and so camera trap design should be planned accordingly. To estimate annual nest abundance for track count foot patrol data, Whiting et al. (2013) created simulation models that showed a five‐to‐seven‐fold greater monitoring effort was needed for longer nesting seasons, which is particularly challenging at remote beaches monitored by opportunistic foot patrol surveys (e.g., Cocos Keeling; Whiting et al. 2014) or aerial surveys (Marsh and Saalfeld 1989). Although snapshot foot patrols provide detailed nesting information and aerial surveys increase spatial coverage, there is minimal temporal coverage to detect changes within the nesting season, for example, shifts in nesting phenology (Hawkes et al. 2007) or between seasons to explore inter‐annual variability (Omeyer et al. 2022). When only part of the nesting beach is surveyed by foot patrols, counts can be extrapolated to the whole suitable nesting area (Mortimer et al. 2020). In the same way, greater temporal coverage is possible using camera traps, and counts can be extrapolated from camera trapped areas to the whole nesting beach. Additionally, a combination of snapshot foot patrol surveys, aerial surveys (via Unmanned Aerial Vehicles (UAV), Rees et al. 2018; aeroplanes, Lauriano et al. 2011; or satellite imagery, Casale and Ceriani 2019) and camera traps could be used to acquire detailed nesting information whilst increasing spatial and temporal coverage.

Thirdly, three images every 30 min taken early in the morning were sufficient to obtain one clear image to detect tracks on most occasions. For any remote image monitoring technique, steps can be taken to capture images with little interference, for example, setting cameras to avoid glare (Madsen et al. 2020) from sunrise as in our study. As recommended for sea turtle population surveys (via ground and aerial surveys), camera traps were set to take images at the same time every day in the early morning when the sun angle is low (Schroeder and Murphy 1999). From the ten cameras set to take images every 30 s, we calculated the number of images and the number of days cameras could operate before reaching storage capacity. Theoretically, cameras could capture an image every 2 h for one year before reaching storage capacity and depleting the battery. An image every 2 h would further increase the chances of obtaining a fog‐free image across the day and could help us understand nesting patterns in relation to time of day and tides in more detail. Given hawksbill turtles in the region typically nest in the day and green turtles nest at night (Diamond 1976; Evans et al. 2022), more frequent images could provide a separation between daytime and nighttime nesting when cameras are set during overlapping nesting periods (e.g., October to February; Mortimer et al. 2020). We recommend assessing the battery life while trialling cameras to capture images every 2 h as the battery may drain quicker on these settings over a longer period.

From camera trap images, we found track counts to be variable along the nesting beach, which highlights the importance of the number of camera traps and placement for future work. Reef and beach geomorphology can influence where a turtle emerges from the sea (Cuevas et al. 2021). For example, at Ascension Island, green turtle emergence locations are likely influenced by the offshore topography (Mortimer 1982). For sites where track distribution is known, the pattern of emergences should be highlighted and incorporated into the design of camera trap studies to avoid overestimations when cameras are set up solely in hotspots and vice versa. Inappropriate camera trap placement at Whipsnade Wild Animal Park in the UK led to underestimating the number of mara ( *Dolichotis patagonum* ) (Rowcliffe et al. 2008). For sites where track distribution is unknown, camera traps can be set up on beaches specifically to evaluate the distribution of emergences. Additionally, cameras can be used to monitor variations in hotspots as reef and beach geomorphology change over time.

In our study, we found that green turtle emergences followed the pattern of increasing around neap tides and decreasing around spring tides. Likewise, Witt et al. (2009) found an increase in nesting effort by leatherbacks during days around neap tides in Gabon, yet peak nesting occurs around spring tides in French Guiana (Girondot and Fretey 1996). Studies of olive ridley ( *Lepidochelys olivacea* ) solitary and arribada nesting events in Costa Rica reported more females emerge during the weaker neap tide phase than any other moon phase (Dornfield et al. 2015; Bézy et al. 2020). Regional differences may be attributed to site characteristics (e.g., topography of the beach or tidal patterns such as diurnal or semidiurnal tides). We highlight the importance of understanding emergence patterns to effectively apply correction factors when designing a camera trap study to estimate nesting females from limited track counts.

For sea turtle census studies, an understanding of track longevity at a study site is important to obtain accurate nesting female numbers and to factor in the number of potentially missed tracks. From camera trap images, we found tracks persisted over the same number of days during spring and neap tides. Mean track longevity from camera traps of 3 days, ranging from 0 to 9 days, was similar but lower than estimates of 4 days, ranging from 1 to 9 days, from foot patrol surveys during another season at our site (Mortimer et al. 2020). Tracks can still be observed into the vegetation on foot patrols, which is out of sight from camera traps, and so we would expect higher track longevity recorded from foot patrols at our study site. Another reason for this slight difference could be seasonality, as estimates from foot patrols were conducted between November and December (Mortimer et al. 2020), and from camera traps between April and September. In the archipelago, moderate winds blow generally from the northwest between October and April, and the strong southeast trade winds blow for the rest of the year (Sheppard et al. 1999) and so we might expect tracks to persist from October to April and disappear quicker between May and September, especially given the Index Beach is located on the southeast coast. Similarly, in Aldabra, Seychelles, green turtle track longevity ranged from 10 days between June and October to 14 days across other months (Gibson 1979). The comparison, along with the discrepancies known between the two methods, validates the use of camera traps to estimate track longevity effectively. The advantage of using camera traps is the ability to assess track longevity across the whole nesting season, covering multiple tidal cycles and weather conditions. Future camera trap studies should aim to estimate track longevity across the two monsoon seasons and by species to confirm these findings.

Our study was conducted over the peak green turtle nesting season, outside of the peak hawksbill nesting season (Mortimer et al. 2020) and so we were confident (supported by foot patrol observations) that all tracks recorded by camera traps were from green turtles. For nesting beaches with multiple species, identifying species from tracks in images (e.g., remote sensing imagery and UAV images, Potter et al. 2018; Wang et al. 2019) adds an element of complexity. Active deep learning systems have been incorporated into camera trap image analysis for terrestrial animal studies, which decrease the time for observers to manually identify animals (Norouzzadeh et al. 2020). Yet, these systems differentiate between animals that are distinct from one another. Green and hawksbill turtle tracks can be distinguished by crawl characteristics and track width on foot patrols, as green turtle tracks are wider (> 100 cm wide) than hawksbills (Mortimer et al. 2020). However, given the angle of the camera traps, it is difficult to obtain positive identification. Stokes et al. (2023) compared species length to width ratios from physical turtle captures and UAV surveys, and the results were similar across the two methods. In a similar approach, nesting seasonality data and foot patrol counts could be used to assign species ratios to tracks in images.

In conclusion, our findings add to the number of studies using camera traps to monitor and estimate animal abundance (e.g., jaguars *Panthera onca* , Silver et al. 2004; ungulates, Taylor et al. 2021; red squirrels *Sciurus vulgaris* ; Shannon et al. 2023). Our results support the use of camera traps to estimate the number of sea turtles nesting at a site where nesting success and clutch frequency are known and contribute to the application of camera traps for sea turtle research. As such, camera traps should be considered for monitoring sea turtle nesting sites where possible when surveys are temporally fragmented, such as remote and inaccessible locations. We encourage the use of camera traps for track counts at beaches of different lengths and nesting densities to further understand the applicability of camera trap surveys for sea turtle nesting beaches around the world.

## Author Contributions

**Holly J. Stokes:** data curation (lead), formal analysis (lead), investigation (equal), methodology (equal), project administration (equal), resources (equal), software (equal), validation (equal), visualization (lead), writing – original draft (lead), writing – review and editing (equal). **Graeme C. Hays:** conceptualization (equal), data curation (supporting), formal analysis (supporting), funding acquisition (equal), investigation (equal), methodology (equal), project administration (equal), resources (equal), software (equal), supervision (equal), validation (equal), visualization (supporting), writing – original draft (supporting), writing – review and editing (equal). **Kimberley L. Stokes:** formal analysis (supporting), validation (supporting), visualization (supporting), writing – review and editing (equal). **Nicole Esteban:** conceptualization (equal), data curation (supporting), formal analysis (supporting), funding acquisition (equal), investigation (equal), methodology (equal), project administration (equal), resources (equal), supervision (equal), validation (equal), visualization (supporting), writing – original draft (supporting), writing – review and editing (equal).

## Conflicts of Interest

The authors declare no conflicts of interest.

## Supporting information


**Appendix S1:** ece372138‐sup‐0001‐AppendixS1‐S2.docx.
**Appendix S2:** ece372138‐sup‐0001‐AppendixS1‐S2.docx.

**Data S1:** ece372138‐sup‐0002‐DataS1.csv.

**Data S2:** ece372138‐sup‐0003‐DataS2.csv.

## Data Availability

The data that support the findings of this study are openly available in Dryad at https://doi.org/10.5061/dryad.6hdr7srd7.

## References

[ece372138-bib-0001] Bézy, V. S. , N. F. Putman , J. A. Umbanhowar , et al. 2020. “Mass‐Nesting Events in Olive Ridley Sea Turtles: Environmental Predictors of Timing and Size.” Animal Behaviour 163: 85–94. 10.1016/j.anbehav.2020.03.002.

[ece372138-bib-0002] Broderick, A. C. , F. Glen , B. J. Godley , and G. C. Hays . 2003. “Variation in Reproductive Output of Marine Turtles.” Journal of Experimental Marine Biology and Ecology 288: 95–109. 10.1016/S0022-0981(03)00003-0.

[ece372138-bib-0003] Casale, P. , and S. A. Ceriani . 2019. “Satellite Surveys: A Novel Approach for Assessing Sea Turtle Nesting Activity and Distribution.” Marine Biology 166: 47. 10.1007/s00227-019-3494-4.

[ece372138-bib-0004] Cuevas, E. , M. de los Ángeles Liceaga‐Correa , E. Nuñez‐Lara , and I. Mariño‐Tapia . 2021. “How Changes in Beach Morphology Affect Hawksbill Turtle ( *Eretmochelys imbricata* ) Nesting Distribution at Celestun, Yucatan, Mexico.” Regional Studies in Marine Science 44: 101714. 10.1016/j.rsma.2021.101714.

[ece372138-bib-0005] Dewald, J. R. , and D. A. Pike . 2013. “Geographical Variation in Hurricane Impacts Among Sea Turtle Populations.” Journal of Biogeography 41: 307–316. 10.1111/jbi.12197.

[ece372138-bib-0006] Diamond, A. W. 1976. “Breeding Biology and Conservation of Hawksbill Turtles, *Eretmochelys imbricata* L., on Cousin Island, Seychelles.” Biological Conservation 9: 199–215. 10.1016/0006-3207(76)90010-0.

[ece372138-bib-0007] Dornfield, T. C. , N. J. Robinson , P. Santidrián Tomillo , and F. V. Paladino . 2015. “Ecology of Solitary Nesting Olive Ridley Sea Turtles at Playa Grande, Costa Rica.” Marine Biology 162: 123–139. 10.1007/s00227-014-2583-7.

[ece372138-bib-0008] Evans, S. , M. J. Schulze , S. Dunlop , et al. 2022. “Investigating the Effectiveness of a Well‐Managed Hatchery as a Tool for Hawksbill Sea Turtles (*Eretmochelys imbricata*) Conservation.” Conservation Science and Practice 4: e12819. 10.1111/csp2.12819.

[ece372138-bib-0009] Fieberg, J. R. , K. Jenkins , S. McCorquodale , C. G. Rice , G. C. White , and K. White . 2015. “Do Capture and Survey Methods Influence Whether Marked Animals Are Representative of Unmarked Animals.” Wildlife Society Bulletin 39: 713–720. 10.1002/wsb.591.

[ece372138-bib-0010] Fisher, J. T. , B. Anholt , and J. P. Volpe . 2011. “Body Mass Explains Characteristic Scales of Habitat Selection in Terrestrial Mammals.” Ecology and Evolution 1: 517–528. 10.1002/ece3.45. 22393519 PMC3287334

[ece372138-bib-0011] Fonseca, L. G. , S. Arroyo‐Arce , I. Thomson , et al. 2020. “Impacts of Jaguar Predation on Nesting Sea Turtles at Nancite Beach, Santa Roda National Park, Costa Rica.” Herpetological Conservation and Biology 15: 547–557.

[ece372138-bib-0012] Fossette, S. , G. Loewenthal , L. R. Peel , et al. 2021. “Using Aerial Photogrammetry to Assess Stock‐Wide Marine Turtle Nesting Distribution, Abundance and Cumulative Exposure to Industrial Activity.” Remote Sensing 13: 1116. 10.3390/rs13061116.

[ece372138-bib-0013] Fuentes, M. M. P. B. , E. McMichael , C. Y. Kot , et al. 2023. “Key Issues in Assessing Threats to Sea Turtles: Knowledge Gaps and Future Directions.” Endangered Species Research 52: 303–341. 10.3354/esr01278.

[ece372138-bib-0014] Gibson, T. S. H. 1979. “Green Turtle (*Chelonia mydas*) Nesting Activity at Aldabra Atoll.” Philosophical Transactions of the Royal Society of London. Series B, Biological Sciences 286: 255–263. 10.1098/rstb.1979.0033.

[ece372138-bib-0015] Girondot, M. 2017. “Optimizing Sampling Design to Infer the Number of Marine Turtles Nesting on Low and High Density Sea Turtle Rookeries Using Convolution of Negative Binomial Distribution.” Ecological Indicators 81: 83–89. 10.1016/j.ecolind.2017.05.063.

[ece372138-bib-0016] Girondot, M. , and J. Fretey . 1996. “Leatherback Turtles, *Dermochelys coriacea*, Nesting in French Guiana, 1978‐1995.” Chelonian Conservation and Biology 2: 204–208.

[ece372138-bib-0017] Gronwald, M. , Q. Genet , and M. Touron . 2019. “Predation on Green Sea Turtle, *Chelonia mydas*, Hatchlings by Invasive Rats.” Pacific Conservation Biology 25: 423–424. 10.1071/PC18087.

[ece372138-bib-0018] Guilder, J. , B. Barca , S. Arroyo‐Arce , R. Gramajo , and R. Salom‐Pérez . 2015. “Jaguars ( *Panthera onca* ) Increase Kill Utilization Rates and Share Prey in Response to Seasonal Fluctuations in Nesting Green Turtle ( *Chelonia mydas* ) Abundance in Tortuguero National Park, Costa Rica.” Mammalian Biology 80: 65–72. 10.1016/j.mambio.2014.11.005.

[ece372138-bib-0019] Hamel, S. , S. T. Killengreen , J.‐A. Henden , et al. 2012. “Towards Good Practice Guidance in Using Camera‐Traps in Ecology: Influence of Sampling Design on Validity of Ecological Inferences.” Methods in Ecology and Evolution 4: 105–113. 10.1111/j.2041-210x.2012.00262.x.

[ece372138-bib-0020] Hawkes, L. A. , A. C. Broderick , M. H. Godfrey , and B. J. Godley . 2007. “Investigating the Potential Impacts of Climate Change on a Marine Turtle Population.” Global Change Biology 13: 923–932. 10.1111/j.1365-2486.2007.01320.x.

[ece372138-bib-0021] Hays, G. C. , L. C. Ferreira , A. M. M. Sequeira , et al. 2016. “Key Questions in Marine Megafauna Movement Ecology.” Trends in Ecology & Evolution 31: 463–475. 10.1016/j.tree.2016.02.015. 26979550

[ece372138-bib-0022] Hays, G. C. , A. D. Mazaris , and G. Schofield . 2022. “Inter‐Annual Variability in Breeding Census Data Across Species and Regions.” Marine Biology 169: 54. 10.1007/s00227-022-04042-x.

[ece372138-bib-0023] Hays, G. C. , G. Schofield , M. Papazekou , A. Chatzimentor , S. Katsanevakis , and A. D. Mazaris . 2024. “A Pulse Check for Trends in Sea Turtle Numbers Across the Globe.” IScience 27: 109071. 10.1016/j.isci.2024.109071. 38524373 PMC10960059

[ece372138-bib-0024] Kays, R. , B. S. Arbogast , M. Baker‐Whatton , et al. 2020. “An Empirical Evaluation of Camera Trap Study Design: How Many, How Long and When?” Methods in Ecology and Evolution 11: 700–713. 10.1111/2041-210X.13370.

[ece372138-bib-0025] Kikukawa, A. , N. Kamezaki , and H. Ota . 2001. “Factors Affecting Nesting Beach Selection by Loggerhead Turtles (*Caretta caretta*): A Multiple Regression Approach.” Journal of Zoology 249: 447–454. 10.1111/j.1469-7998.1999.tb01214.x.

[ece372138-bib-0026] Labonne, J. , and P. Gaudin . 2005. “Exploring Population Dynamics Patterns in a Rare Fish, *Zingel aper*, Through Capture‐Mark‐Recapture Methods.” Conservation Biology 19: 463–472. 10.1111/j.1523-1739.2005.00013.x.

[ece372138-bib-0027] Lamont, M. M. , D. Ingram , T. Baker , M. Weigel , and B. M. Shamblin . 2023. “Confirmation of Significant Sea Turtle Nesting Activity on a Remote Island Chain in the Gulf of Mexico.” Ecology and Evolution 13: e10448. 10.1002/ece3.10448. 37608924 PMC10441180

[ece372138-bib-0028] Lasala, J. A. , M. C. Macksey , K. T. Mazzarella , K. L. Main , J. J. Foote , and A. D. Tucker . 2023. “Forty Years of Monitoring Increasing Sea Turtle Relative Abundance in the Gulf of Mexico.” Scientific Reports 13: 17213. 10.1038/s41598-023-43651-4. 37821522 PMC10567714

[ece372138-bib-0029] Lauriano, G. , S. Panigada , P. Casale , N. Pierantonio , and G. P. Donovan . 2011. “Aerial Survey Abundance Estimates of the Loggerhead Sea Turtle *Caretta caretta* in the Pelagos Sanctuary, Northwestern Mediterranean Sea.” Marine Ecology Progress Series 437: 291–302. 10.3354/meps09261.

[ece372138-bib-0030] Lei, J. , and D. T. Booth . 2017. “Who Are the Important Predators of Sea Turtle Nests at Wreck Rock Beach?” PeerJ 5: e3515. 10.7717/peerj.3515. 28674666 PMC5494172

[ece372138-bib-0031] Lovemore, T. E. J. , N. Montero , S. A. Ceriani , and M. M. P. B. Fuentes . 2020. “Assessing the Effectiveness of Different Sea Turtle Nest Protection Strategies Against Coyotes.” Journal of Experimental Marine Biology and Ecology 533: 151470. 10.1016/j.jembe.2020.151470.

[ece372138-bib-0032] Luo, G. , W. Wei , Q. Dai , and J. Ran . 2020. “Density Estimation of Unmarked Populations Using Camera Traps in Heterogeneous Space.” Wildlife Society Bulletin 44: 173–181. 10.1002/wsb.1060.

[ece372138-bib-0033] Lyet, A. , S. Waller , T. Chambert , et al. 2023. “Estimating Animal Density Using the Space‐To‐Event Model and Bootstrap Resampling With Motion‐Triggered Camera‐Trap Data.” Remote Sensing in Ecology and Conservation 10: 141–155. 10.1002/rse2.361.

[ece372138-bib-0034] Madsen, A. E. , L. Corral , and J. J. Fontaine . 2020. “Weather and Exposure Period Affect Coyote Detection at Camera Traps.” Wildlife Society Bulletin 44: 342–350. 10.1002/wsb.1080.

[ece372138-bib-0035] Marsh, H. , and W. K. Saalfeld . 1989. “Aerial Surveys of Sea Turtles in the Northern Great Barrier Reef Marine Park.” Wildlife Research 16: 239–249. 10.1071/WR9890239.

[ece372138-bib-0036] Mazaris, A. D. , G. Schofield , C. Gkazinou , V. Almpanidou , and G. C. Hays . 2017. “Global Sea Turtle Conservation Successes.” Science Advances 3: e1600730. 10.1126/sciadv.1600730. 28948215 PMC5606703

[ece372138-bib-0037] McCallum, J. 2013. “Changing Use of Camera Traps in Mammalian Field Research: Habitats, Taxa and Study Types.” Mammal Review 43: 196–206. 10.1111/j.1365-2907.2012.00216.x.

[ece372138-bib-0038] McCarthy, E. D. , J. M. Martin , M. M. Boer , and J. A. Welbergen . 2022. “Ground‐Based Counting Methods Underestimate True Numbers of a Threatened Colonial Mammal: An Evaluation Using UAV‐Based Thermal Surveys as a Reference.” Wildlife Research 50: 484–493. 10.1071/WR21120.

[ece372138-bib-0039] McDonald, L. L. 2004. “Sampling Rare Populations.” In Sampling Rare or Elusive Species: Concepts, Designs, and Techniques for Estimating Population Parameters, edited by W. L. Thompson , 11–42. Island Press.

[ece372138-bib-0040] Medeiros, L. , M. Chaloupka , A. B. Bolten , et al. 2022. “Tracking Green Turtle Nesting Trends at a Remote Oceanic Rookery.” Marine Biology 169: 86. 10.1007/s00227-022-04054-7.

[ece372138-bib-0041] Mortimer, J. A. 1982. “Factors Influencing Beach Selection by Nesting Sea Turtles.” In Biology and Conservation of Sea Turtles, edited by K. A. Bjorndal , 45–52. Smithsonian Institute Press.

[ece372138-bib-0042] Mortimer, J. A. , N. Esteban , A. N. Guzman , and G. C. Hays . 2020. “Estimates of Marine Turtle Nesting Populations in the South‐West Indian Ocean Indicate the Importance of the Chagos Archipelago.” Oryx 54: 332–343. 10.1017/S0030605319001108.

[ece372138-bib-0043] Mrosovsky, N. , P. H. Dutton , and C. P. Whitmore . 1984. “Sex Ratios of Two Species of Sea Turtle Nesting in Suriname.” Canadian Journal of Zoology 62: 2227–2239. 10.1139/z84-324.

[ece372138-bib-0044] Nichols, J. D. 2014. “The Role of Abundance Estimates in Conservation Decision‐Making.” In Applied Ecology and Human Dimensions in Biological Conservation, edited by L. Verdade , M. Lyra‐Jorge , and C. Piña , 117–131. Springer. 10.1007/978-3-642-54751-5_8.

[ece372138-bib-0045] Norouzzadeh, M. S. , D. Morris , S. Beery , N. Joshi , N. Jojic , and J. Clune . 2020. “A Deep Active Learning System for Species Identification and Counting in Camera Trap Images.” Methods in Ecology and Evolution 12: 150–161. 10.1111/2041-210X.13504.

[ece372138-bib-0046] Omeyer, L. C. M. , T. J. McKinley , N. Bréheret , et al. 2022. “Missing Data in Sea Turtle Population Monitoring: A Bayesian Statistical Framework Accounting for Incomplete Sampling.” Frontiers in Marine Science 9: 817014. 10.3389/fmars.2022.817014.

[ece372138-bib-0049] Pauli, J. N. , J. P. Whiteman , M. D. Riley , and A. D. Middleton . 2010. “Defining Non‐Invasive Approaches for Sampling Vertebrates.” Conservation Biology 24: 349–352. 10.1111/j.1523-1739.2009.01298.x. 19624526

[ece372138-bib-0050] Piacenza, S. E. H. , J. R. Piacenza , K. J. Faller II , N. J. Robinson , and T. R. Siegfried . 2022. “Design and Fabrication of a Stereo‐Video Camera Equipped Unoccupied Aerial Vehicle for Measuring Sea Turtles, Sharks, and Other Marine Fauna.” PLoS One 17: e0276382. 10.1371/journal.pone.0276382. 36256654 PMC9578584

[ece372138-bib-0051] Potter, L. C. , C. J. Brady , and B. P. Murphy . 2018. “Accuracy of Identifications of Mammal Species From Camera Trap Images: A Northern Australian Case Study.” Austral Ecology 44: 473–483. 10.1111/aec.12681.

[ece372138-bib-0052] Pritchard, P. C. H. , and J. A. Mortimer . 1999. “Taxonomy, External Morphology, and Species Identification.” In Research and Management Techniques for the Conservation of Sea Turtles, edited by K. L. Eckert , K. A. Bjorndal , F. A. Abreu‐Grobois , and M. Donnelly , 16–17. IUCN/SSC Marine Turtle Specialist Group.

[ece372138-bib-0053] R Core Team . 2024. R: A Language and Environment for Statistical Computing. R Foundation for Statistical Computing. https://www.R‐project.org/.

[ece372138-bib-0054] Rahman, D. A. , and A. A. A. F. Rahman . 2021. “Performance of Unmanned Aerial Vehicle With Thermal Imaging, Camera Trap, and Transect Survey for Monitoring of Wildlife.” IOP Conference Series: Earth and Environmental Science 771: 12011. 10.1088/1755-1315/771/1/012011.

[ece372138-bib-0055] Rees, A. F. , L. Avens , K. Ballorain , et al. 2018. “The Potential of Unmanned Aerial Systems for Sea Turtle Research and Conservation: A Review and Future Directions.” Endangered Species Research 35: 81–100. 10.3354/meps09261.

[ece372138-bib-0056] Rowcliffe, J. M. , J. Field , S. T. Turvey , and C. Carbone . 2008. “Estimating Animal Density Using Camera Traps Without the Need for Individual Recognition.” Journal of Applied Ecology 45: 1228–1236. 10.1111/j.1365-2664.2008.01473.x.

[ece372138-bib-0057] Rowcliffe, J. M. , R. Kays , B. Kranstauber , C. Carbone , and P. A. Jansen . 2014. “Quantifying Levels of Animal Activity Using Camera Trap Data.” Methods in Ecology and Evolution 5: 1170–1179. 10.1111/2041-210X.12278.

[ece372138-bib-0058] Royle, J. A. , K. U. Karanth , A. M. Gopalaswamy , and N. S. Kumar . 2009. “Bayesian Inference in Camera Trapping Studies for a Class of Spatial Capture‐Recapture Models.” Ecology 90: 3233–3244. 10.1890/08-1481.1. 19967878

[ece372138-bib-0059] Schroeder, B. , and S. Murphy . 1999. “Population Surveys (Ground and Aerial) on Nesting Beaches.” In Research and Management Techniques for the Conservation of Sea Turtles, edited by K. L. Eckert , K. A. Bjorndal , F. A. Abreu‐Grobois , and M. Donnelly , 45–55. IUCN/SSC Marine Turtle Specialist Group.

[ece372138-bib-0060] Shannon, G. , S. Valle , and C. M. Shuttleworth . 2023. “Capturing Red Squirrels (*Sciurus vulgaris*) on Camera: A Cost‐Effective Approach for Monitoring Relative Abundance and Habitat Preference.” Ecology and Evolution 13: e10536. 10.1002/ece3.10536. 37794876 PMC10546084

[ece372138-bib-0061] Sheppard, C. R. C. , M. R. D. Seaward , R. Klaus , and J. M. W. Topp . 1999. “The Chagos Archipelago: An Introduction.” In Ecology of the Chagos Archipelago, edited by C. R. C. Sheppard and M. R. D. Seaward , 1–20. Linnean Society Occasional Publishing 2. Westbury Publishing.

[ece372138-bib-0062] Shimada, T. , M. G. Meekan , R. Baldwin , et al. 2021. “Distribution and Temporal Trends in the Abundance of Nesting Sea Turtles in the Red Sea.” Biological Conservation 261: 109235. 10.1016/j.biocon.2021.109235.

[ece372138-bib-0063] Silver, S. C. , L. E. T. Ostro , L. K. Marsh , et al. 2004. “The Use of Camera Traps for Estimating Jaguar *Panthera onca* Abundance and Density Using Capture/Recapture Analysis.” Oryx 38: 148–154. 10.1017/S0030605304000286.

[ece372138-bib-0064] Sims, M. , R. Bjorkland , P. Mason , and L. B. Crowder . 2008. “Statistical Power and Sea Turtle Nesting Beach Surveys: How Long and When?” Biological Conservation 141: 2921–2931. 10.1016/j.biocon.2008.07.021.

[ece372138-bib-0065] Southwell, C. , C. G. M. Paxton , D. Borchers , P. Boveng , T. Rogers , and W. K. de la Mare . 2008. “Uncommon or Cryptic? Challenges in Estimating Leopard Seal Abundance by Conventional but State‐Of‐The‐Art Methods.” Deep Sea Research Part I: Oceanographic Research Papers 55: 519–531. 10.1016/j.dsr.2008.01.005.

[ece372138-bib-0079] Stokes, H. J. , J. A. Mortimer , J‐O. Laloë , G. C. Hays , and N. Esteban . 2023. “Synergistic Use of UAV Surveys, Satellite Tracking Data, and Mark‐Recapture to Estimate Abundance of Elusive Species.” Ecosphere 14: e4444. 10.1002/ecs2.4444.

[ece372138-bib-0066] Taylor, J. C. , S. B. Bates , J. C. Whiting , B. R. McMillan , and R. T. Larsen . 2021. “Using Camera Traps to Estimate Ungulate Abundance: A Comparison of Mark‐Resight Methods.” Remote Sensing in Ecology and Conservation 8: 32–44. 10.1002/rse2.226.

[ece372138-bib-0067] Tucker, A. D. , K. L. Pendoley , K. Murray , et al. 2021. “Regional Ranking of Marine Turtle Nesting in Remote Western Australia by Integrating Traditional Ecological Knowledge and Remote Sensing.” Remote Sensing 13: 4696. 10.3390/rs13224696.

[ece372138-bib-0068] Udevitz, M. S. , C. V. Jay , and M. B. Cody . 2005. “Observer Variability in Pinniped Counts: Ground‐Based Enumeration of Walruses at Haul‐Out Sites.” Marine Mammal Science 21: 108–120. 10.1111/j.1748-7692.2005.tb01211.x.

[ece372138-bib-0069] Wang, D. , Q. Shao , and H. Yue . 2019. “Surveying Wild Animals From Satellites, Manned Aircraft and Unmanned Aerial Systems (UASs): A Review.” Remote Sensing 11: 1308. 10.3390/rs11111308.

[ece372138-bib-0070] Weber, S. B. , N. Weber , J. Ellick , et al. 2014. “Recovery of the South Atlantic's Largest Green Turtle Nesting Population.” Biodiversity and Conservation 23: 3005–3018. 10.1007/s10531-014-0759-6.

[ece372138-bib-0071] Welbourne, D. J. , A. W. Claridge , D. J. Paull , and F. Ford . 2020. “Camera‐Traps Are a Cost‐Effective Method for Surveying Terrestrial Squamates: A Comparison With Artificial Refuges and Pitfall Traps.” PLoS One 15: e0226913. 10.1371/journal.pone.0226913. 31945104 PMC6964851

[ece372138-bib-0072] Whiting, A. U. , M. Chaloupka , and C. J. Limpus . 2013. “Comparing Sampling Effort and Errors in Abundance Estimates Between Short and Protracted Nesting Seasons for Sea Turtles.” Journal of Experimental Marine Biology and Ecology 449: 165–170. 10.1016/j.jembe.2013.09.016.

[ece372138-bib-0073] Whiting, A. U. , M. Chaloupka , and C. J. Limpus . 2020. “Sampling Nesting Sea Turtles: Impact of Survey Error on Trend Detection.” Marine Ecology Progress Series 634: 213–223. 10.3354/meps13202.

[ece372138-bib-0074] Whiting, A. U. , M. Chaloupka , N. Pilcher , P. Basintal , and C. J. Limpus . 2021. “Sampling Nesting Sea Turtles: Optimizing Survey Design to Minimize Error.” Marine Ecology Progress Series 674: 257–270. 10.3354/meps13824.

[ece372138-bib-0075] Whiting, S. D. , I. Macrae , R. Thorn , W. Murray , and A. U. Whiting . 2014. “Sea Turtles of the Cocos (Keeling) Islands, Indian Ocean.” Raffles Bulletin of Zoology 30: 168–183.

[ece372138-bib-0076] Whiting, S. D. , and A. U. Whiting . 2011. “Predation by the Saltwater Crocodile ( *Crocodylus porosus* ) on Sea Turtle Adults, Eggs, and Hatchlings.” Chelonian Conservation and Biology 10: 198–205. 10.2744/CCB-0881.1.

[ece372138-bib-0077] Witt, M. J. , B. Baert , A. C. Broderick , et al. 2009. “Aerial Surveying of the World's Largest Leatherback Turtle Rookery: A More Effective Methodology for Large‐Scale Monitoring.” Biological Conservation 142: 1719–1727. 10.1016/j.biocon.2009.03.009.

[ece372138-bib-0078] Zwerts, J. A. , P. J. Stephenson , F. Maisels , et al. 2021. “Methods for Wildlife Monitoring in Tropical Forests: Comparing Human Observations, Camera Traps, and Passive Acoustic Sensors.” Conservation Science and Practice 3: e568. 10.1111/csp2.568.

