# Supplementary material for: Camera Traps: A Novel Method to Estimate Numbers of Nesting Sea Turtles
Source: Ecol Evol. 2025 Sep 15;15(9):e72138. doi: 10.1002/ece3.72138 (PMC12434351; doi:10.1002/ece3.72138)
Supplement: Supplementary file 1 — Appendix S1: ece372138‐sup‐0001‐AppendixS1‐S2.docx. Appendix S2: ece372138‐sup‐0001‐AppendixS1‐S2.docx. [file ECE3-15-e72138-s002.docx]

**Appendix 1**

A pilot study was conducted between 2018-2019 using camera trap models (e.g., Ereagle Wildlife Trail Camera 12MP 1080P, Campark Trail Game Camera 12MP 1080P) to test image quality and robustness. Apeman H70 (30MP 4K; *n* = 13; with 32GB memory cards and lithium-ion AA batteries) trail cameras were selected for this study and attached to vegetation (e.g., *Cocos nucifera*; *Tournefortia argentea*; Figure A1a) with a releasable cable tie (HellermannTyton, 580 (L) x 28mm (W), 360N Tensile Strength, SOFTFIX Series). Cameras were positioned to capture the length of beach where possible or slightly towards the sea depending on vegetation obstruction and from each camera, a ‘turtle track’ was marked in the sand at 5 m interval (Figure A1b).


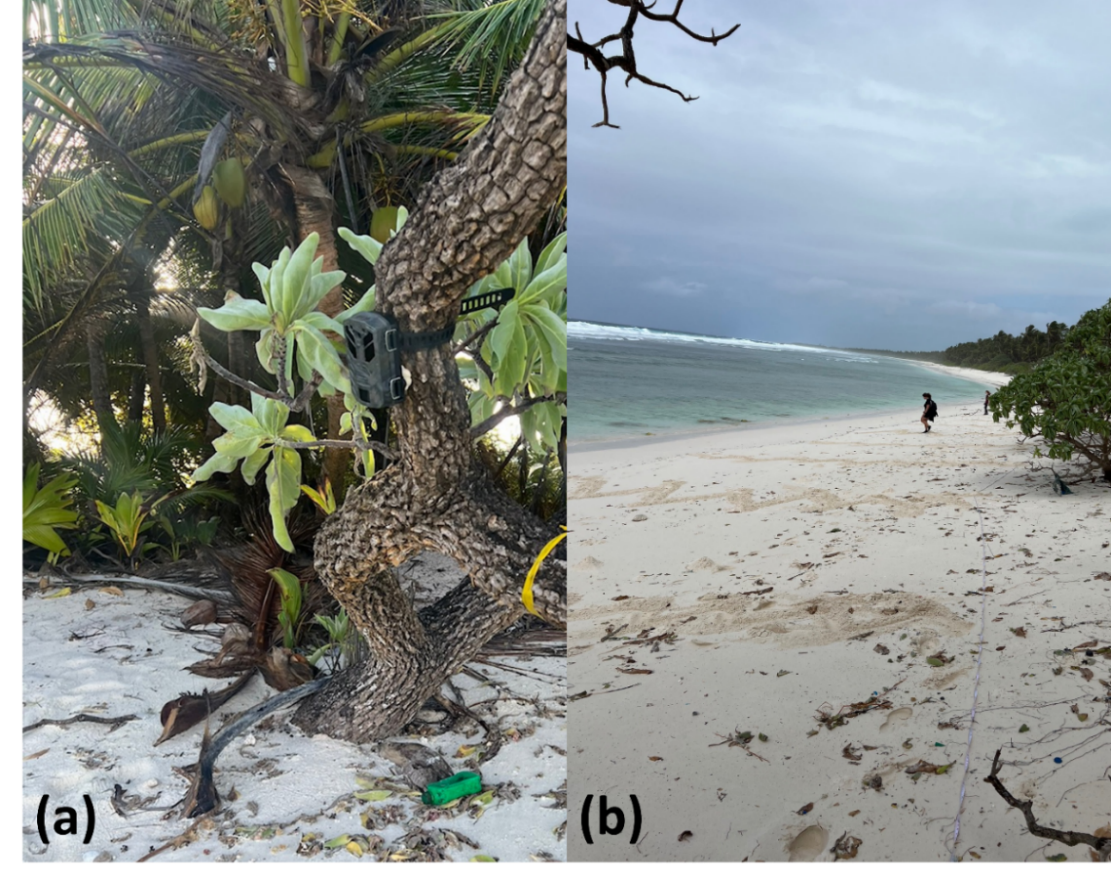


**Figure A1. a)** A camera trap (Apeman H70 trail camera, 30MP, 4K) set up on a beach heliotrope (*Tournefortia* *argentea*) tree using a releasable cable tie (HellermannTyton, 580 (L) x 28mm (W), 360N Tensile Strength, SOFTFIX Series) on the Index Beach in Diego Garcia, Chagos Archipelago. **b)** Image of ‘turtle tracks’ created in the sand at 5m intervals to determine the observable distance from each camera trap.

**Table A1**. Estimated mean distance (± SD and range) of beach coverage by each camera trap along the Index Beach, Diego Garcia, Chagos Archipelago.

| **Camera ID** | **Estimated distance observed (m)** | |
| --- | --- | --- |
|  | **Mean ± SD** | **Range** |
| CT1 | 30 ± 0 | 30-30 |
| CT2 | 15 ± 7 | 5-20 |
| CT3 | 8 ± 4 | 5-15 |
| CT4 | 10 ± 0 | 10-10 |
| CT5 | 15 ± 0 | 15-15 |
| CT6 | 9 ± 2 | 5-15 |
| CT7 | 12 ± 5 | 5-15 |
| CT8 | 8 ± 5 | 5-15 |
| CT9 | 5 ± 0 | 5-5 |
| CT10 | 7 ± 3 | 5-10 |
| CT11 | 10 ± 0 | 10-10 |
| CT12 | 10 ± 7 | 5-20 |
| CT13 | 8 ± 6 | 5-20 |

**Appendix 2**

A log-log plot of camera trap beach coverage and track count variability showed a clear straight-line relationship (log(SD) = 0.90724-0.47902*log(Coverage); *R^2^* = 0.99; *F_1,4_* = 1443; *p* < 0.0001), confirming a power-law relationship between camera trap coverage and the variability in the mean number of tracks per day over a 90-d simulation (Figure A2).

**
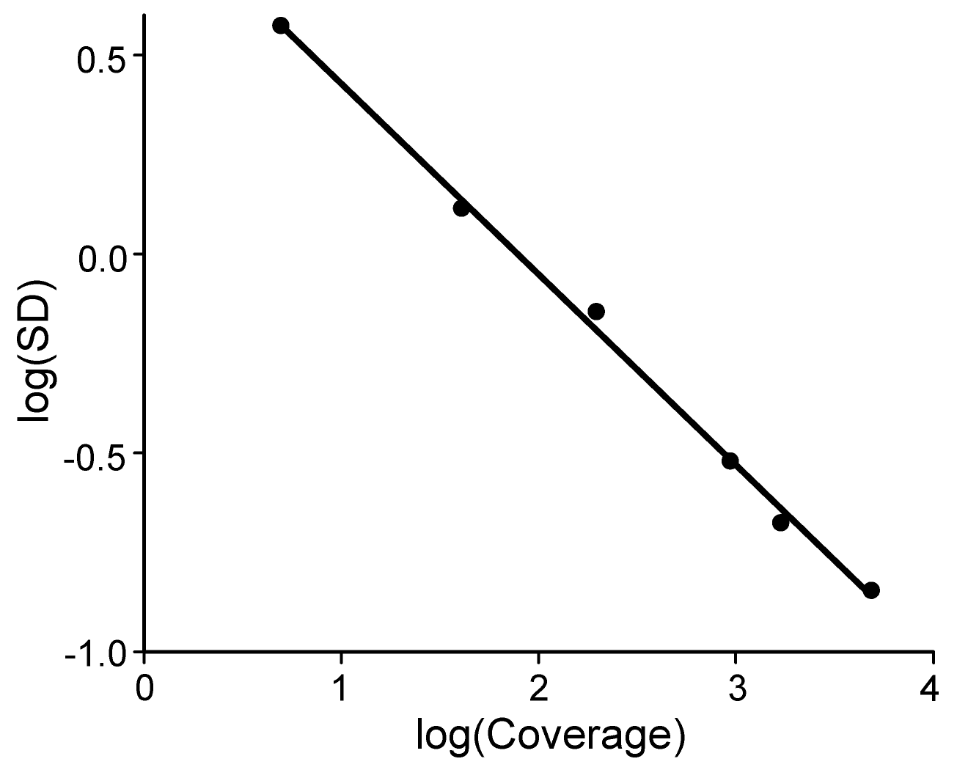
**

**Figure A2.** Log-log plot of beach coverage (%) vs. mean track count variability (SD, tracks per day) showing a strong straight-line relationship, where variability decreases as coverage increases (log(SD) = 0.90724-0.47902*log(Coverage); *R^2^* = 0.99; *F_1,4_* = 1443; *p* < 0.0001). The straight-line relationship on logged scales suggests a power-law relationship between camera trap beach coverage and the extent of variability in the mean number of tracks per day over a 90-d simulation.
